# Ultrahard stitching of nanotwinned diamond and cubic boron nitride in C_2_-BN composite

**DOI:** 10.1038/srep30518

**Published:** 2016-07-27

**Authors:** Xiaobing Liu, Xin Chen, Hong-An Ma, Xiaopeng Jia, Jinsong Wu, Tony Yu, Yanbin Wang, Jiangang Guo, Sylvain Petitgirard, Craig R. Bina, Steven D. Jacobsen

**Affiliations:** 1 Department of Earth and Planetary Sciences, Northwestern University, Evanston, Illinois 60208, USA; 2 State Key Laboratory of Superhard Materials, Jilin University, Changchun, Jilin 130012, China; 3 Northwestern University Atomic and Nanoscale Characterization Experimental (NUANCE) center, Northwestern University, Evanston, Illinois 60208, USA; 4 Center for Advanced Radiation Sources, University of Chicago, Chicago, Illinois 60439, USA; 5 Department of Physics & Astronomy, Rice University, Houston, Texas 77005-1827, USA; 6 Bayerisches Geoinstitut, University of Bayreuth, Bayreuth 95444, Germany

## Abstract

Materials combining the hardness and strength of diamond with the higher thermal stability of cubic boron nitride (*c*BN) have broad potential value in science and engineering. Reacting nanodiamond with *c*BN at moderate pressures and high temperatures provides a pathway to such materials. Here we report the fabrication of C_*x*_-BN nanocomposites, measuring up to 10 mm in longest dimension, by reacting nanodiamond with pre-synthesized *c*BN in a large-volume press. The nanocomposites consist of randomly-oriented diamond and *c*BN domains stitched together by *sp*^3^-hybridized C-B and C-N bonds, leading to *p*-type semiconductivity. Dislocations near the sutures accommodate lattice mismatch between diamond and *c*BN. Nanotwinning within both diamond and *c*BN domains further contributes to a bulk hardness ~50% higher than sintered *c*BN. The nanocomposite of C_2_-BN exhibits *p*-type semiconductivity with low activation energy and high thermal stability, making it a functional, ultrahard substance.

Superhard and ultrahard materials, presently defined as having Vickers hardness (*H*_*V*_) greater than 40 and 80 GPa, respectively, are sought after for their potential use as extreme abrasives and other applications in high-pressure science and technology^1^,^2^,^3^,^4^. Diamond is generally considered the hardest known substance, but due to graphitization in air at 800–900 K and reactivity with transition metals at high temperature, diamond has limited application in certain grinding environments. Cubic boron nitride (*c*BN), a diamond-structured compound, is widely used as an abrasive with much higher thermal stability than diamond (~1473 K) and low reactivity with steel, but the Vickers hardness of *c*BN (40–60 GPa)^5^,^6^ is only about half that of diamond. Because of their similar ionic radii, B, C, and N may form diamond-like compounds in solid solution that are expected to be ultrahard with higher thermal and chemical stability than diamond^7^,^8^,^9^,^10^,^11^,^12^. Based on theoretical predictions, experimental studies have attempted to synthesize a number of superhard B-C-N materials including diamond-like BC_2_N^13^, BC_4_N^10^, BC_3_ 14 and BC_5_ 15,16. However, solid-solution B-C-N phases are generally considered metastable because syntheses usually lead to segregated carbon and boron compounds^17^. The extremely high pressures (20–25 GPa) and temperatures (2000–2500 K) of many B-C-N syntheses ultimately limit production potentials and sample size^7^,^15^,^17^.

Recently, it was discovered that nanotwinned (nt) diamond^3^ and nt-*c*BN^2^,^18^ possess ultrahigh hardness and toughness, mitigating the so-called reverse Hall-Petch effect by shear strengthening from compressive forces across nanospaced twin boundaries^18^. Combining nt-diamond and nt-*c*BN to create an ultrahard nanocomposite would potentially lead to a material with optimized properties of each. Reacting diamond and *c*BN at high pressure and high temperature (HPHT) has been the typical route to synthesizing composites of diamond and cubic boron nitride (C_*x*_-BN)^19^,^20^,^21^,^22^,^23^, where *x* is the proportion of diamond relative to *c*BN. The conditions and mechanisms of stitching diamond and *c*BN together remains the primary challenge. Recently, an epitaxial *c*BN/diamond heterojunction was produced by growing single-crystal *c*BN on diamond seed crystals using a temperature gradient method^24^. A network of continuous stacking faults, arranged by hexagonal dislocation loops on the {111} heterointerface was found to accommodate the *c*BN/diamond lattice mismatch (~1.4 Å)^24^. In this study, we fabricated highly uniform C_*x*_-BN (*x* = 2, 4, 6, 8) nanocomposites at 7.5 GPa and 2273 K, which consist of randomly oriented, ~50 nm domains of diamond and *c*BN (~250 nm). The bulk samples, measuring up to 10-mm diameter by 5-mm in height, combine several novel features from recent syntheses in the diamond-*c*BN system^2^,^3^,^18^,^24^; in particular, the new C_2_-BN composite possesses nanotwinning in both diamond and *c*BN domains, which are stitched by *sp*^3^-hybridized C-B and C-N bonds along randomly-oriented interfaces. Near the sutures, dislocations accommodate the lattice mismatch between diamond and *c*BN and, as recently predicted^24^, the C_2_-BN nanocomposite is a *p*-type semiconductor with low activation energy. Among the suite of new nanocomposites, C_2_-BN is the hardest, with *Hv* = 85(2) GPa, placing it in the ultrahard class of materials.

We control the stoichiometry in C_*x*_-BN, by varying the proportions of starting powders of diamond (average grain size 50 nm) and *c*BN (average grain size 250 nm). Synthesis was conducted at 7.5 GPa while holding constant temperature of 2273 K for two hours (Table 1). The components of C_2_-BN remain diamond-structured; however, the recovered C_*x*_-BN composites with C_4_-BN, C_6_-BN, and C_8_-BN contained diamond and *c*BN together with appearance of graphitic phase (Fig. S1). The presence of minor amounts of graphite decreases with increasing ratio of *c*BN added to the starting material, indicating that *c*BN plays a role as catalyst in stitching diamond and *c*BN domains while protecting the diamond from graphitization at the high temperatures of synthesis (2273 K). Since the aim of this study is to synthesize and characterize nanopolycrystalline C_*x*_-BN composite free of graphite, here we focus on the physical properties of C_2_-BN, which was the only sample not exhibiting the presence of graphite.

Figure 1a shows a representative X-ray diffraction (XRD) pattern of the recovered C_2_-BN composite. With increasing synthesis temperature, the diffraction lines become broader and the peaks of diamond and *c*BN from the 2273 K synthesis have considerable overlap (Fig. S1). Raman spectra (Fig. 1b) of C_2_-BN produced at 7.5 GPa and 2273 K are dominated by one sharp Raman peak at 1332 cm^−1^ and two weak peaks at about 1055 and 1305 cm^−1^, corresponding to diamond and *c*BN longitudinal optical and transverse optical modes, respectively. The Fourier transform infrared (FTIR) spectrum (Fig. S2) shows two main peaks at 1065 and 1318 cm^−1^ together with one weak shoulder at 1208 cm^−1^, which correspond to stretching of the *sp*^3^ B-N bonds in *c*BN^21^,^22^, C-N bonds within the sutures of diamond and *c*BN domains^25^ and C-C bonds in diamond^26^, respectively. This characterization shows that the C_2_-BN samples consist of diamond and *c*BN domains, consistent with XRD data (Fig. 1a). The recovered samples from 7.5 GPa and 1500–2273 K were black and opaque, while transparent samples were achieved by subsequent annealing at 15–18 GPa and 2100 K (Fig. S3).

Scanning electron microscopy (SEM) confirms the C_2_-BN composite is uniform and well sintered (Fig. 2a). Thin foils of C_2_-BN synthesized at 7.5 GPa and 2273 K were prepared by focused-ion beam (FIB) techniques for transmission electron microscopy (TEM). Characteristic TEM and high-resolution TEM (HRTEM) images are shown in Fig. 2b–e. The TEM images in Fig. 2b,c show the absence of porosity in the C_2_-BN sample, unlike many sintered diamond samples from which catalysts have been removed. The samples exhibit a nanopolycrystalline structure, consisting of hybridized, randomly distributed diamond and *c*BN domains (Figs S4 and S5). Furthermore, HRTEM images reveal that nanotwinned structures have been produced during HPHT treatment (Fig. 2d,e). These multiple twins in the diamond and *c*BN domains are 1.5–2 nm and 6–8 nm wide, respectively. HRTEM images in Fig. 3 show that the two main domains of diamond and *c*BN have been stitched together by high-pressure sintering. The rigid lattice misfit between diamond and *c*BN domains is accommodated by the presence of partial dislocations and stacking faults at the interface between the two domains (Fig. S6)^24^. The sutures between diamond-*c*BN domains are typically 1–2 nm thick. HRTEM images and selected area electron diffraction (SAED) patterns (Fig. 3c,d) from the diamond-*c*BN junctions reveal that *c*BN domains twin to conjoin the adjacent diamond. Stacking faults and dislocations are observed in *c*BN in close proximity to the interfaces (Fig. S6). Through this misfit accommodation mechanism^24^, strain between diamond and *c*BN domains is released, leading to stability of the C_2_-BN composite.

To determine the local bonding characteristics across the diamond-*c*BN sutures, we carried out inelastic X-ray scattering (IXS; beam size 20 μm) and electron energy-loss spectroscopy (EELS; beam size 1 nm) on the C_2_-BN sample. The IXS spectra in Fig. S7 show that all three elements, B, C and N, are *sp*^3^ hybridized along the sutures. In contrast, pure diamond sintered under identical conditions (7.5 GPa and 2273 K) exhibits both *sp*^2^ and *sp*^3^ bonds (Fig. S8). This confirms that graphitization of sintered diamond can be effectively minimized or eliminated in C_2_-BN by choosing the optimal synthesis conditions. EELS was conducted to analyze the interface between diamond and *c*BN from the selected area in the C_2_-BN sample from 2273 K (Fig. 4a). Figure 4b shows the EELS spectrum and all the three bonds corresponding to the characteristic *K*-shell ionization edges of B, C and N in the diamond-*c*BN interface. These EELS edge structures prove that bonding within the sutures are primarily *sp*^3^-hybridized B-C-N bonds together with small amount of *sp*^2^ C-B bonds.

X-ray photoelectron spectroscopy (XPS) was performed to study the stoichiometry of the hybridized diamond-*c*BN junctions. Figures 4c–e show XPS spectra of B, N and C, respectively. The shape and position of the spectra for all three elements are different from previous studies on pure diamond^27^, *c*BN^28^, and B-C-N compounds^29^,^30^. The C 1*s* peak is located at 285.2 eV (Fig. 4c), which is similar to the value of C 1*s* in *sp*^3^ C-C bonds observed in diamond^31^. The full width at half-maximum (FWHM) is about 2.3 eV, suggesting different valence states of carbon are present. Two smaller peaks at higher (285.9 eV) and lower (284.5 eV) binding energy are due to C 1*s* in *sp*^3^ C-N and C-B bonds, respectively^32^. In addition, a higher binding energy at 289.0 eV is observed from the contribution of C 1*s* in C-O bonds^29^,^33^. The main peak of the B 1*s* spectrum is located at 190.7 eV, very close to that of B 1*s* (191.0 eV) in pure *c*BN^28^,^29^, suggesting the main bonding configuration in the C_2_-BN composite produced at 7.5 GPa and 2273 K is similar to that of *c*BN, where one N atom is surrounded by four B atoms. However, a small shoulder at lower binding energy implies a contribution of B and C, because of the lower electronegativity of C atoms than N^34^. A higher binding energy at 192.4 eV implies a contribution from the configuration of B 1*s* in B-O bonds^35^. The N 1*s* binding energy is located at 398.4 eV (Fig. 4e), similar to the position of N 1*s* in *c*BN^28^,^29^. However, two small shoulders are observed at higher binding energy of 400.3 and 402.1 eV, which are due to N 1*s* in C-N and C-O, respectively^29^,^30^. All the C 1*s*, B 1*s* and N 1*s* spectra indicate that the main configuration for C, B and N atoms is *sp*^3^ B-N bonding, together with a contribution from the bonding configuration in the boundary region between diamond and *c*BN domains. In addition to *sp*^3^-hybridized C-N and C-B bonds produced during HPHT reaction, some C-O, B-O, and N-O bonds remain from the starting materials exposed to air.

Vickers hardness measurements were conducted on the C_*x*_-BN nanocomposites using a standard square-pyramidal diamond indenter. We imaged and compared the indented surfaces using an optical microscope, SEM, and well-calibrated 3D optical microscope to determine the *H*_*V*_ value. Figure 5a shows the hardness-load curve of C_2_-BN. The asymptotic region is reached at loads above 4.9 N and shows an ultrahard value of 82(3) GPa at 19.6 N. In a second C_2_-BN sample, the indentation at 19.6 N load corresponded to *H*_*V*_ = 85(2) GPa, confirming the ultrahard value of our nanocomposite C_2_-BN. For other compositions of C_*x*_-BN as well as *c*BN and sintered diamond, we studied indentations at 19.6 N load and found a peak hardness for the composition C_2_-BN (Fig. 5b). *H*_V_ of the sintered diamond sample is lower than sintered *c*BN because of the presence of a graphitic phase on the diamond surface (Fig. S8).

The compressibility of C_2_-BN was studied under quasi-hydrostatic pressures up to 40 GPa at 300 K in a diamond-anvil cell using synchrotron X-ray diffraction at Sector 16 (HPCAT) of the Advanced Photon Source (APS). Figure S10 shows the compression data of diamond and *c*BN domains in the bulk C_2_-BN sample, together with the previously reported equations of state of single-crystal diamond^36^ and ultrahard nanopolycrystalline (NP) *c*BN^17^. The *P*-*V* data (Table S2) were fitted to a third-order Vinet equation of state, finding for diamond *V*_0_ = 45.33 (±0.01) Å^3^ and *K*_T0_ = 436.4 (±2.3) GPa when *K*_0_’ = 3.0 is fixed to the value from a previous study^36^ to allow for better comparison of *K*_T0_. Our value of *K*_T0_ is close to the adiabatic bulk modulus derived from recent ultrasonic measurements, *K*_T0_ = 441.8 (±0.8) for single-crystal diamond and *K*_T0_ = 442.5 (±0.5) GPa for NP-diamond^37^. For the *c*BN domains, we obtained *V*_0_ = 47.30 (±0.01) Å^3^ and *K*_T0_ = 398.6 (±2.5) GPa when *K*_0_’ = 2.3 is fixed to the value of a previous study^17^,^38^. When *K*_0_’ is allowed to refine, we obtain *V*_0_ = 47.33 (±0.01) Å^3^, *K*_T0_ = 377.4 (±7.1) GPa, and *K*_0_’ = 3.6 (±0.4) (Fig. S10). Using *K*_T0_ = 398.6 (±2.5) GPa for the *c*BN domains, we obtain a theoretical nanopolycrystalline average *K*_T0_ value of the C_2_-BN composite of 417.5 (±2.4) GPa.

We also determined the thermal stability (in air) of the C_*x*_-BN samples by thermoanalytical analysis (TG/DTG) from 400 K to 1620 K (Fig. 5c). The synthetic C_2_-BN sample shows remarkably higher stability than either single-crystal boron-doped diamond (~1000 ppm) with the onset temperature of oxidation in air, T_OX_ ~1027 K, or nanopolycrystalline diamond (T_OX_ ~950 K)^17^. The synthetic nanocomposite of C_2_-BN remains stable up to 1183 K. Moreover, the oxidation rate for C_2_-BN composite decreases substantially between 1183 K and 1425 K.

To investigate the electrical properties of our synthetic C_*x*_-BN nanocomposites, Hall measurements were performed with four electrodes at room temperatures (Table 1). The sintered diamond (sample S6) and C_*x*_-BN materials with high carbon concentration (sample S5) show good *n*-type conductivity with a low resistivity that can be due to the graphitization under HPHT treatment. Both C_2_-BN and C_4_-BN nanocomposites synthesized at 2273 K and 7.5 GPa exhibit *p*-type semiconductivity. Resistivity of the *p*-type C_*x*_-BN is observed to decrease and the carrier concentration increases with increasing synthetic temperature condition up to 2273 K (sample S9 and S10). The *p*-type C_*x*_-BN synthesized at 2273 K (sample S2-4) shows low electrical resistivity, and the electron and hole mobility is in a wide range of 2.7–365 cm^2^ V^−1^ s^−1^. The carrier concentration of the C_2_-BN composite can reach 1.4387 × 10^19 ^cm^−3^ at 300 K. This value is comparable with that of B-doped diamond^39^. These results suggest that we can control the electrical properties of our C_*x*_-BN materials from insulator to highly conducting by adjusting synthetic *P-T* or tuning the ratio between the C and BN with high flexibility and applications in electronics.

Temperature-dependent electrical measurements on our C_2_-BN nanocomposite (Fig. 5d) show a significant increase in resistance by more than 7-fold on cooling from room temperature (ρ = 21Ω) to 1.8 K, a typical semiconducting behavior. The value of the activation energy is calculated on the basis of a linear Arrhenius plot of the logarithm of the resistance ln (*R*) versus the inverse temperature *T*^−1^ in the temperature range between from 100 K and 50 K (See Fig. 5d, inset). The calculated activation energy is 6.21 meV and is similar to hybridized BN-C graphene^30^. Since both pure diamond and *c*BN are good insulators, the *p*-type semiconductivity of the C_*x*_-BN composite must derive from the *sp*^3^-hybridized C-B and some B-B bonds within the sutures of diamond and *c*BN domains^30^,^40^.

This study introduces a new strategy and direction in the search for novel conductive ultrahard materials. Nanocomposite C_*x*_-BN materials will enable the design of particular physical properties in diamond-based structures. Previously, B-doping has been the most effective method used to control the electrical properties of diamond. However, it is difficult to synthesize high-quality diamond crystals with heavy B-doping because boron is typically heterogeneously distributed within the crystal and it is difficult to avoid graphitic defects^41^,^42^. The current synthesis and characterization of *p*-type bulk nanopolycrystalline C_*x*_-BN with low activation energy suggests that stable and uniform diamond-based semiconductors can be formed in large quantities and in a reproducible way. In addition, the well-sintered nanopolycrystalline bulk pieces possess no cleavage, which will allow C_*x*_-BN composite materials to be fabricated into arbitrary shapes for industrial applications such as machine parts. Combining the high thermal conductivity and radiation resistance of diamond with the toughness of *c*BN at high temperatures, the ultrahard C_*x*_-BN bulk nanocomposites have potential applications in electronics under extreme thermal and pressure conditions.

In summary, we synthesized C_*x*_-BN composites with dimensions up to 10 mm × 5 mm at 7.5 GPa and 2273 K. The composite of nanotwinned diamond and *c*BN in a 2:1 ratio (C_2_-BN) shows ultrahard properties by Vickers indentation tests, low compressibility, high thermal stability, and *p*-type semiconductivity - a combination of features that have not previously been found together in pure diamond-like B-C-N materials. Subsequent annealing at 15–18 GPa and 2100 K improves its transparency. The nanotwinned domains of diamond and *c*BN are stitched by *sp*^3^-hybridized C-B and C-N bonds with dislocations near the grain boundaries to accommodate the rigid lattice mismatch of diamond and *c*BN, thus combining the features of a variety of recently reported ultrahard materials^1^,^2^,^3^,^18^,^23^,^24^. The moderate synthesis *P*-*T* conditions and rapid annealing time (2 hours) to form ultrahard nanopolycrystalline C_2_BN composite make it a promising material for machine tooling with other practical benefits. The unique combination of properties in C_2_-BN provides a pathway for fabrication of diamond-based electronics applicable to multifunctional devices operating in extreme environments.

## Methods

### Sample synthesis and characterization

HP-HT synthesis experiments were carried out using a China-type cubic high-pressure apparatus (SPD-6×1200) at the State Key Laboratory of Superhard Materials, Jilin University. High-purity mixtures were prepared of nanodiamond (50 nm) and *c*BN (250 nm) with varying ratios (*x*) of diamond to *c*BN (i.e. C_*x*_-BN), including BN, C_2_-BN, C_4_-BN, C_6_-BN, C_8_-BN, C_10_-BN, C_12_-BN and C. The starting materials were prepared by mechanically mixing for five hours at ambient conditions and treated by hot sulfuric acid/diluted hydrochloric acid to remove impurities introduced during the milling progress. Synthesis temperatures were measured using a Pt-30% Rh/Pt-6%Rh thermocouple junction placed within 0.5 mm of the sample. Pressure was pre-calibrated at high temperatures by the graphite-diamond transition with different catalysts. Samples were first subjected to pressures of 7.5 GPa, heated to temperatures of 1500–2273 K with holding times of 2 hours before rapidly cooling to room temperature in 2 minutes. The C_*x*_-BN cylinders (10-mm diameter × 5-mm height) are black in color. One sample of C_2_-BN was re-annealed at 15–18 GPa and 2100 K for 2 hours using the 1000-ton multi-anvil press at GSECARS (Sector 13) of the APS, Argonne National Laboratory (ANL), producing a transparent and colorless run product (Fig. S3). Run products were initially studied using an optical microscope, SEM, a powder XRD diffractometer, and Raman spectroscopy. Then we employed HRTEM, EELS, IXS, and XPS measurements on the produced C_*x*_-BN composites to determine their chemical compositions and crystal structures.

### Hardness and thermal stability measurement

**Figure 5:**
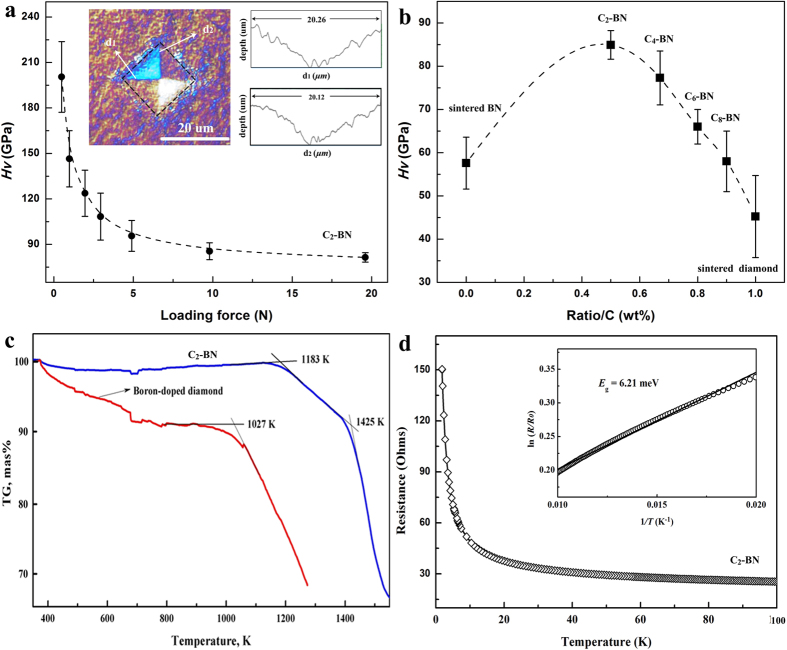
Mechanical and electrical properties of C_2_-BN nanocomposite. **(a)** *H*_*v*_ of C_2_-BN as a function of applied load. Left inset: Optical micrograph of an indentation in C_2_-BN produced by a load of 19.6 N. Right inset images in **(a)** show profiles across d_1_ and d_2_ measured using a 3D microscope. **(b)** *H*_*v*_ as a function of composition for C_*x*_-BN materials. For comparison, we measured the Vickers hardness of C_*x*_-BN with higher diamond:*c*BN ratios at only the highest load of 19.6 N. The measured indentation hardness of C_*x*_-BN ranged from 40 GPa to 85 GPa. **(c)**, Thermogravimetric data (in air) for C_2_-BN (the onset temperature of oxidation *T*_*ox*_ is 1183 K) and boron-doped diamond single crystals (*T*_*ox*_~1027 K). **(d)** A resistance-versus-temperature curve for C_2_-BN measuring 2 × 2 mm and 1 mm thick. The inset in (**d)** shows ln (*R*) as a function of *T*^−1^ in the temperature range from 50 to 100 K. The linear fit (solid line) shows that the data are well described by *R*(*T*) ∝ exp(*E*_g_/*k*_B_*T*), where *E*_g_ is the activation energy and *k*_B_ is Boltzmann’s constant.

Vickers Hardness measurements were performed in the Optical Microscopy & Metallography (OMM) Facility at Northwestern University. A microhardness tester (KB 5 BVZ) was used to measure *H*_*V*_ with a diamond Vickers indenter. *H*_*V*_ was determined from *H*_*V*_ = 1,854.4*F*/*d*^2^, where *F* (in Newtons) is the applied load and *d* (in μm) is the arithmetic mean of the two diagonals (*d*_1_ and *d*_2_) of the Vickers indentation (Fig. 5a). For C_2_-BN, the average of at least seven hardness data points for each load value was used to determine the *H*_V_ from the asymptotic intercept. To study the effect of cracking around the projected area on the inferred indentation dimensions, we used a combination of optical microscopy, SEM, and a high-resolution 3D microscope (Bruker, ContourGT Optical Profiler) to analyze the indented surface. The dynamic TG/DTG studies were performed in air using the NETZSCH STA 449 C thermoanalyser operating at a heating rate of 10 K min^−1^ in the temperature range from 200 to 1650 K. These bulk samples were crushed into powders of micron sizes before the thermoanalytical studies.

### Compressibility

The compressibility measurement was carried out in diamond-anvil cell using neon as the pressure medium. High-pressure X-ray diffraction patterns were collected up to 40 GPa, with exposure times of 5–10 min, at room temperature at beamline 16BM-D (HPCAT) of the APS. The beam size was ~5 × 10 μm at FWHM. Pressure was measured using the ruby fluorescence method^43^. A fragment of C_2_-BN (50 μm diameter, 8–10 μm thick) was placed into the 160-μm diameter hole of a rhenium gasket, pre-indented to 25 μm thickness.

### Electrical measurement

The van der Pauw method was used for electrical transport measurements, in which four electrodes were used in the resistivity measurements. The electrical characteristics were measured with a transport properties measuring system (East Changing ET 9000 Series). The resistivity measurements were carried out at room temperature (300 K) and normal humidity level (40% RH). The temperature dependence of the resistance was measured using a standard four-probe method (PPMS, Quantum Design).

## Additional Information

**How to cite this article**: Liu, X. *et al*. Ultrahard stitching of nanotwinned diamond and cubic boron nitride in C_2_-BN composite. *Sci. Rep.* **6**, 30518; doi: 10.1038/srep30518 (2016).

## Supplementary Material

Supplementary Information

## Figures and Tables

**Figure 1:**
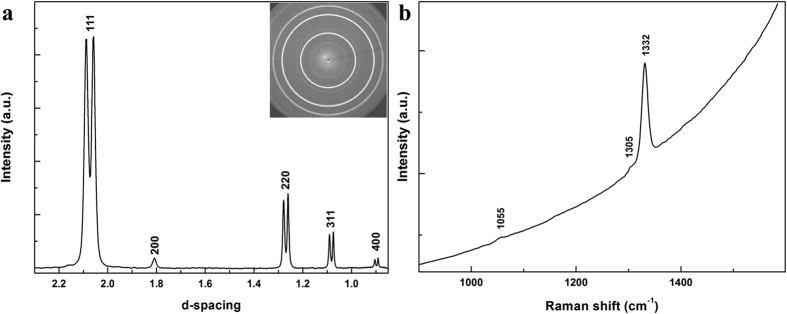
Characterization of C_2_-BN synthesized at 7.5 GPa and 2273 K. (**a**) XRD and (**b**) Raman spectra indicate two components, diamond and *c*BN. Rings in the CCD image (top-right inset in 1a) show the polycrystalline texture of well-sintered C_2_-BN composite.

**Figure 2:**
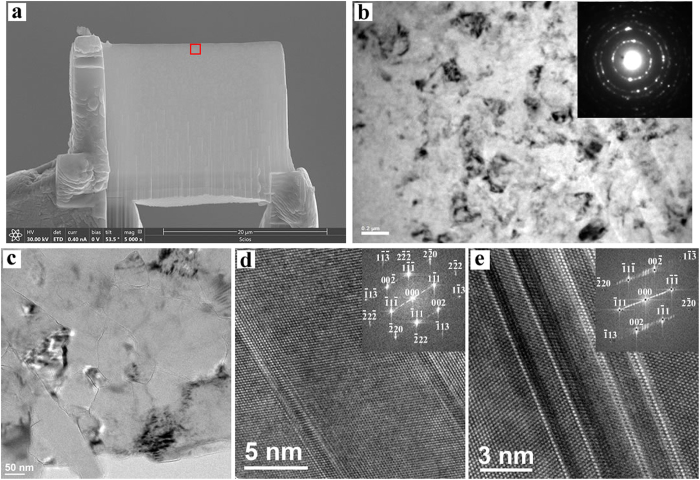
Microstructure of nanocomposite C_2_-BN. **(a)** TEM lamellae obtained from a C_2_-BN bulk sample before focused ion-beam (FIB) thinning. (**b,c)** TEM images of the area with the red box in (**a)**. The inset of (**b)** shows a selected area diffraction (SAED) pattern (**d** and **e**) HRTEM images of the nanotwinned diamond and *c*BN domains. Insets show calculated FFT patterns of the two main domains.

**Figure 3:**
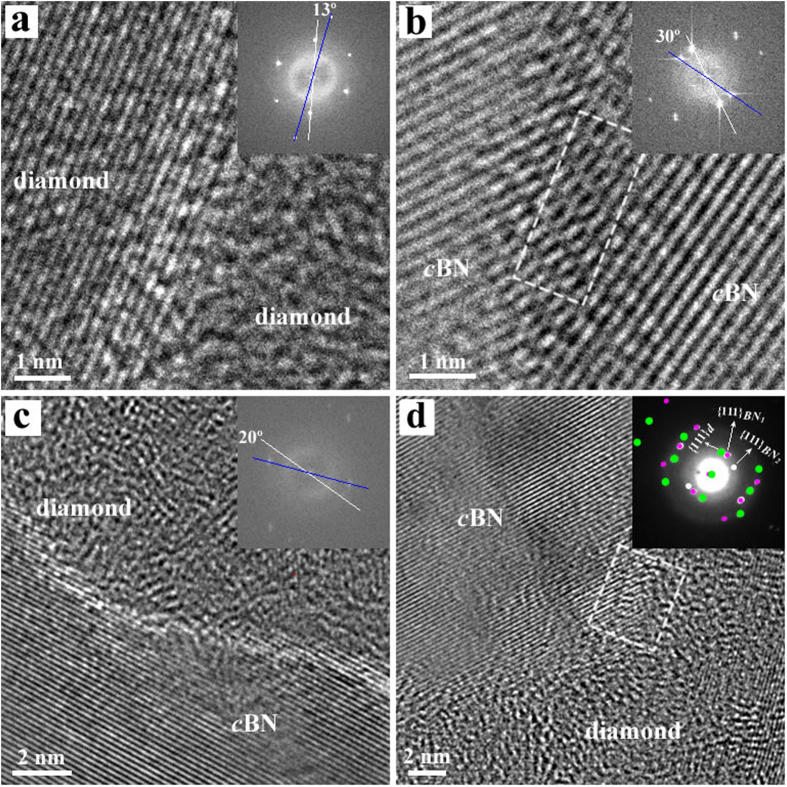
Ultrahard sutures in nanocomposite C_2_-BN. **(a–c)** HRTEM images at the junction between diamond and *c*BN domains. The FFT inset shows the rotation angles between adjacent diamond or *c*BN domains. (**d**) HRTEM and corresponding SAED pattern from a typical junction indicates that only nano-twinned diamond and *c*BN are present at the interface. Dislocations and stacking faults at the interface are found in the region marked by the white rectangle in (**b)** and (**d)**.

**Figure 4:**
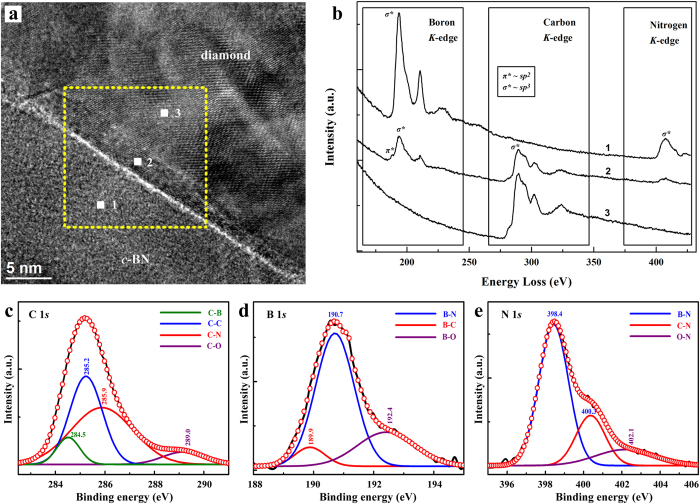
Evidence for *sp*^3^-hybridized C-B and C-N in nanocomposite C_2_-BN. (**a)** HRTEM image of C_2_-BN (**b)** EELS taken from C_2_-BN along the suture between diamond and *c*BN domains. EELS data were collected with a beam approximately 1 × 1 nm in size. Symbols π* and σ* correspond to *sp*^2^ and *sp*^3^ bonding, respectively. (**c–e)** XPS spectra of B, C, and N 1*s* core levels, respectively. The spectra (red circles) are deconvolved (colored curves) by Gaussian fitting.

**Table 1:** Synthesis conditions and electrical properties of nanopolycrystalline C_*x*_-BN composites.

Run	Sample	P (GPa)	T (K)	*p/n*	Resistivity (Ωcm)	Hall mobility (cm^2^/VS)	Carrier concentration (cm^−3^)
**S-1**	Sintered-BN	7.5	2273	/	/	/	/
**S-2**	C_2_-BN	7.5	2273	*p*	1.652	37.55	1.006E17
**S-3**	C_4_-BN	7.5	2273	*p*	0.162	2.67	1.439E19
**S-4**	C_6_-BN	7.5	2273	*p*	0.571	364.69	2.999E16
**S-5**	C_8_-BN	7.5	2273	*n*	0.055	46.62	2.426E18
**S-6**	Sintered-C	7.5	2273	*n*	0.014	15.68	2.749E19
**S-7**	Single-crystal diamond	5-5.5	1500	/	/	/	/
**S-8**	Single-crystal *c*BN	4.8	1400	/	/	/	/
Microcrystalline samples							
**S-9**	C_2_-BN (microcrystalline)	7.5	2273	*p*	6.38	6.55	1.490 E16
**S-10**	C_2_-BN (microcrystalline)	7.5	1800	*p*	1064	162	3.624 E14

Temperature was held constant for two hours. The C_*x*_-BN composites consist of sintered nanodiamond (50 nm) and *c*BN (250 nm) with varying ratios (*x*) of diamond to *c*BN. For comparison, microcrystalline C_2_-BN composites from two different synthesis temperatures demonstrate the decrease in resistivity with increasing temperature between 1800 and 2273 K.
