# Supplementary material for: Ultrahard stitching of nanotwinned diamond and cubic boron nitride in C2-BN composite
Source: Sci Rep. 2016 Jul 27;6:30518. doi: 10.1038/srep30518 (PMC4962311; doi:10.1038/srep30518)
Supplement: Supplementary Information [file srep30518-s1.doc]

**Supplementary Materials**

Ultrahard stitching of nanotwinned diamond and cubic boron nitride in C_2_-BN composite

Xiaobing Liu^1*^, Xin Chen^1^, Hong-An Ma^2^, Xiaopeng Jia^2^, Jinsong Wu^3^, Tony Yu^4^, Yanbin Wang^4^, Jiangang Guo^5^, Sylvain Petitgirard^6^, Craig R. Bina^1^ and Steven D. Jacobsen^1^

*^1^Department of Earth and Planetary Sciences, Northwestern University, Evanston, Illinois 60208, USA*

*^2^State Key Laboratory of Superhard Materials, Jilin University, Changchun, Jilin 130012, China*

*^3^Northwestern University Atomic and Nanoscale Characterization Experimental (NUANCE) center, Northwestern University, Evanston, Illinois 60208, USA*

*^4^Center for Advanced Radiation Sources, University of Chicago, Chicago, Illinois 60439, USA*

*^5^Department of Physics & Astronomy, Rice University, Houston, Texas 77005-1827, USA*

*^6^Bayerisches Geoinstitut, University of Bayreuth, Bayreuth 95444, Germany*

*E-mail: [xiaobing@earth.northwestern.edu](mailto:xiaobing@earth.northwestern.edu)


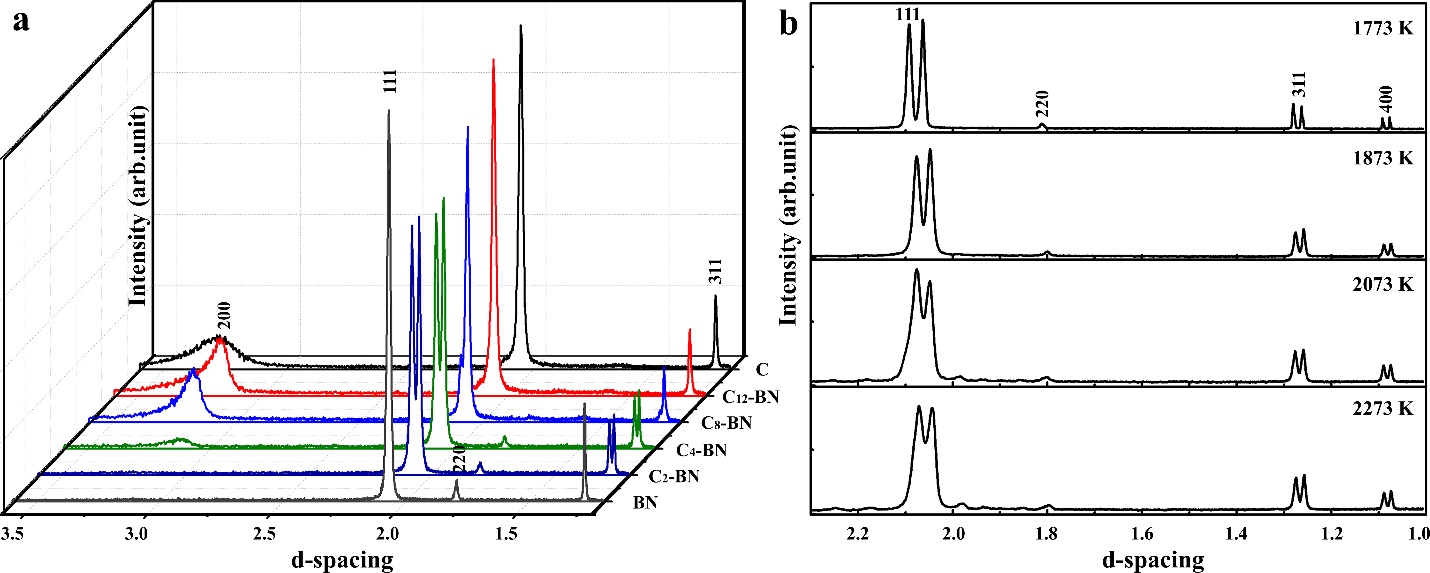


**Figure S1. XRD data for nanocomposite C*_x_*-BN. (a)** XRD patterns of C*_x_*-BN synthesized at 7.5 GPa and 2273 K from Table 1. The recovered C*_x_*-BN samples with diamond to *c*BN ratios higher than 2 (i.e., C_4_-BN, C_6_-BN, C_8_-BN, and C_12_-BN) contained diamond and *c*BN together with appearance of graphitic phase, whereas only cubic phases appear in the C_2_-BN. **(b)** X-ray diffraction patterns of C_2_-BN samples synthesized at 7.5 GPa and temperature range from 1773 K to 2273 K. Peak splitting from segregated phases in diamond + *c*BN composite is observed for the C_2_-BN samples, but varies with temperature because of increased B-C-N hybridization along the sutures between diamond and *c*BN domains.


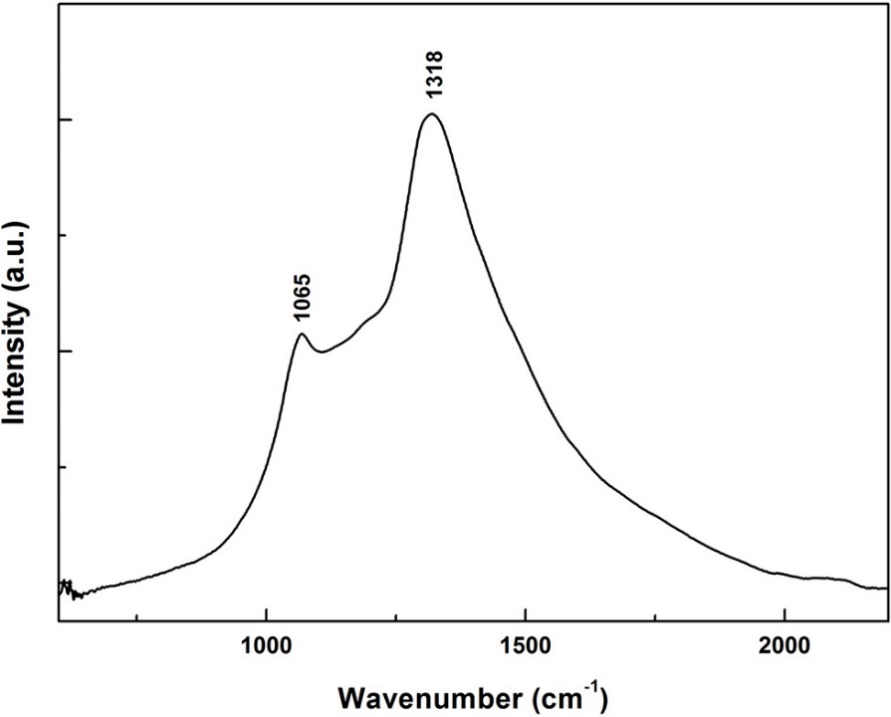


**Figure S2. FTIR spectrum of nanopolycrystalline C_2_-BN composite.** The IR spectrum shows two main peaks at 1065 and 1318 cm^-1^ with a weak shoulder at ~1208 cm^-1^, corresponding to *sp*^3^ B-N bonds in *c*BN [Zhang *et al.* 2004; Zhang *et al.* 2003], C-N bonds within the sutures of diamond and cBN domains [Liu et al. 2014], C-C bonds in diamond [Ahmed *et al.* 2013], respectively.


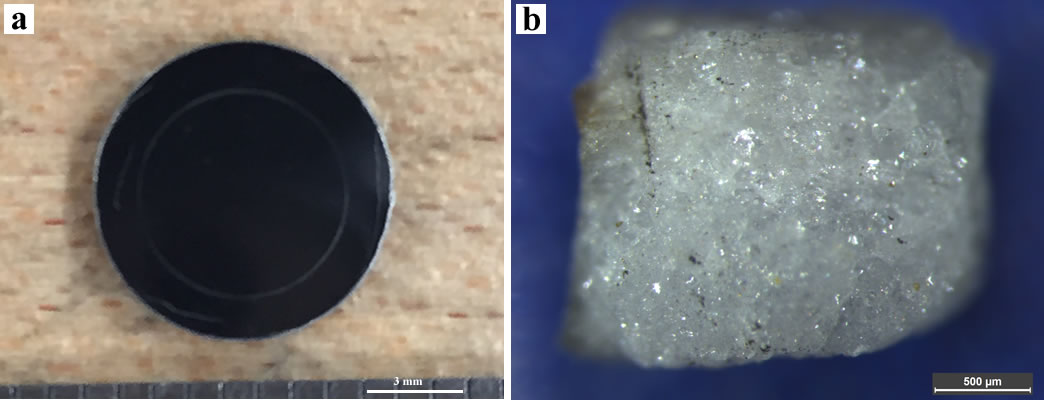


**Figure S3. Optical photomicrographs of nanopolycrystalline C_2_-BN composite.** **(a)** Following synthesis at 7.5 GPa and 2237 K for two hours. **(b)** fragment of a C_2_-BN sample that was re-annealed and 18 GPa and 2100 K for 2 hours became more transparent.


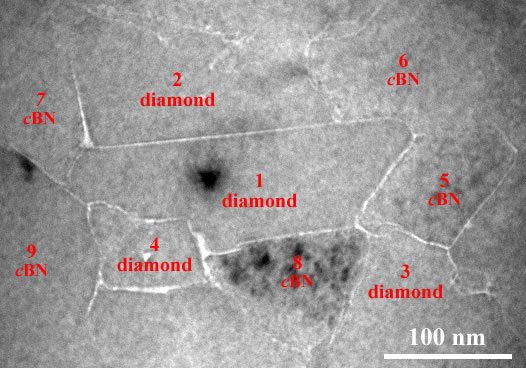


**Figure S4. Characteristic TEM image of nanopolycrystalline C_2_-BN composite.**


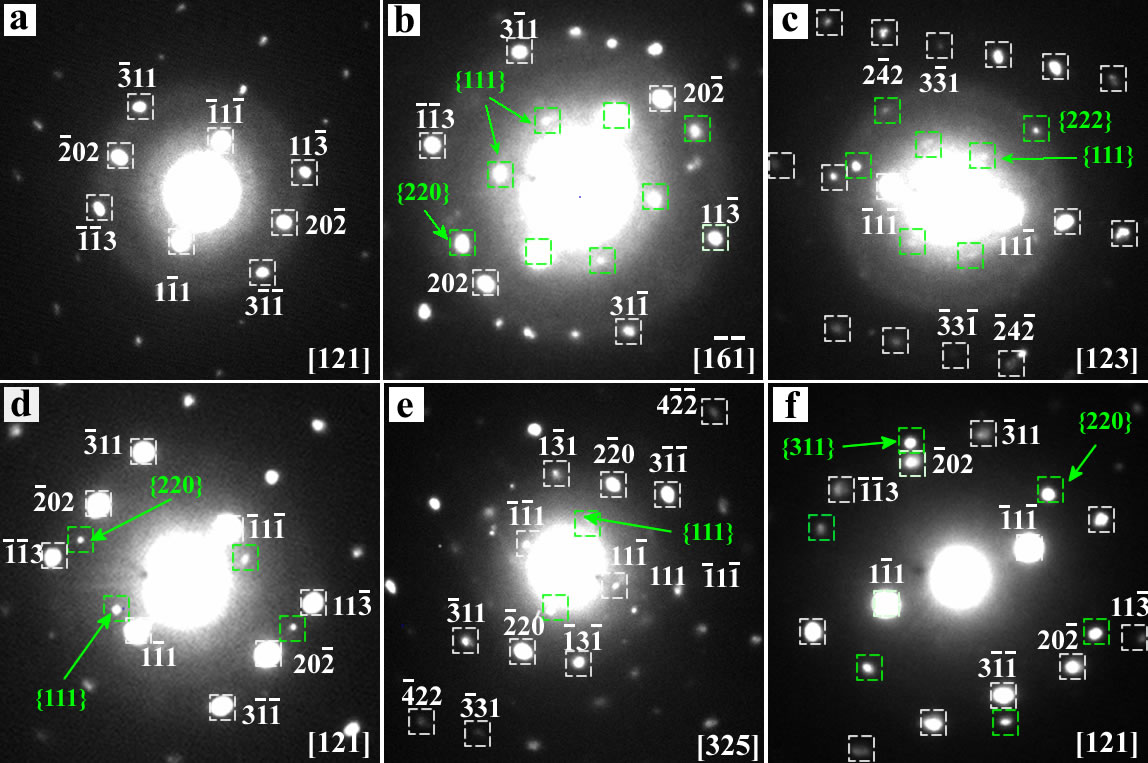


**Figure S5. Corresponding SAED patterns from diamond and *c*BN domains in nanopolycrsytalline C_2_-BN composite.** **(a-c)** SAED patterns from diamond domains. **(d-f)** SAED patterns from *c*BN domains shown in the Fig. S4. The diffraction spots enclosed in green squares result from overlapping diamond or *c*BN domains.


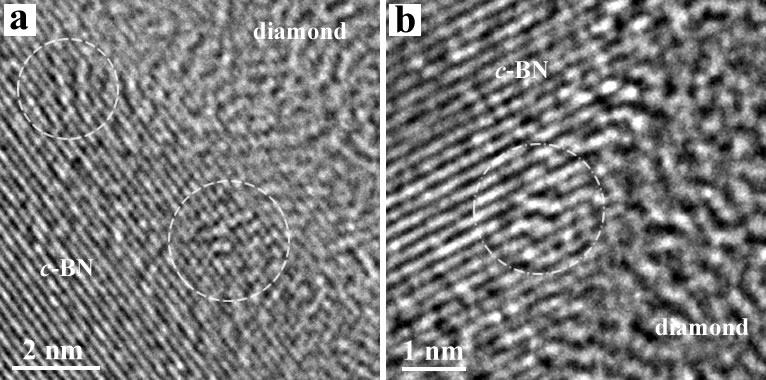


**Figure S6. Dislocations and stacking faults at the interface between diamond and *c*BN domains in nanopolycrystalline C_2_-BN composite.**


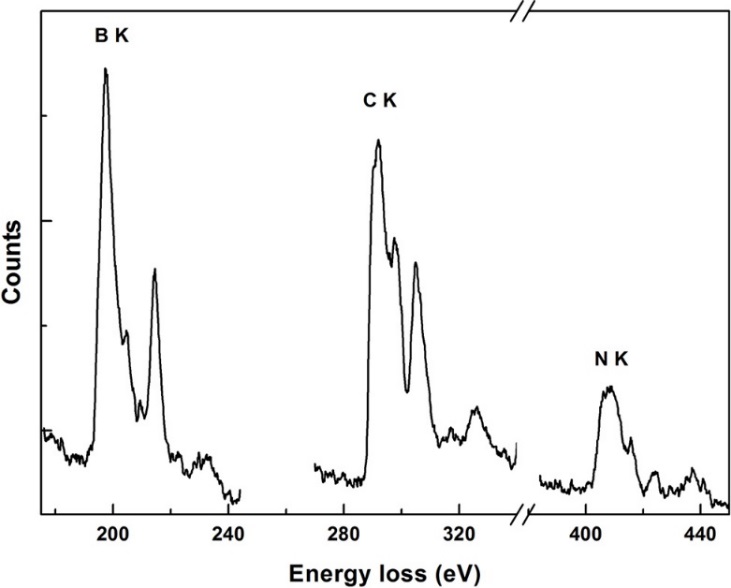


**Figure S7. IXS spectrum of nanopolycrystalline C_2_-BN composite.** IXS measurements on C_2_-BN were conducted with an electron beam focused to 20-µm diameter. The IXS spectrum shows edges starting at 193.7, 287.9 and 403 eV, corresponding to the characteristic K-shell ionization edges of B, C and N, respectively. Only *σ** peaks, which correspond to *sp*^3^ bonds, are observed in the IXS spectra of C_2_-BN.


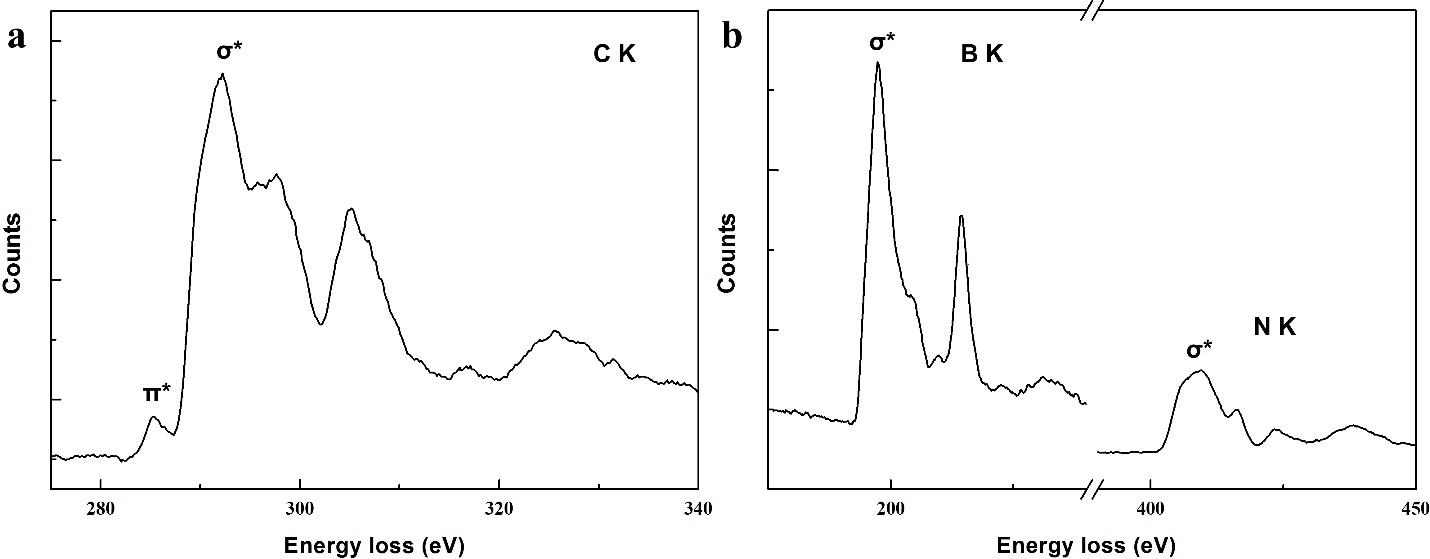


**Figure S8. IXS spectrum of sintered diamond (a) and *c*BN (b), synthesized at 7.5 GPa and 2273 K.** In the sintered diamond spectrum (a), edge structure at 283 eV indicates the presence of *sp*^2^ bonding (*π**) due to minor amount of graphitization, also observed in XRD patterns (Fig. S1).


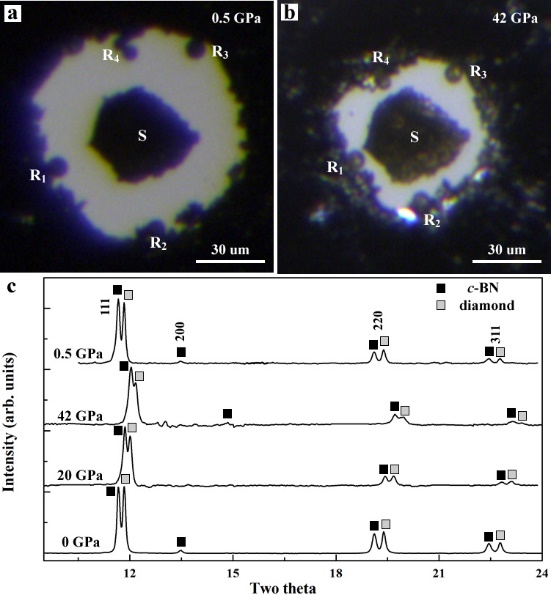


**Figure S9. High-pressure XRD diffraction data. (a, b)** optical photomicrographs of the C_2_-BN samples before and after compression to 42 GPa. **(c)** Diffraction patterns of C_2_-BN as a function of pressure.


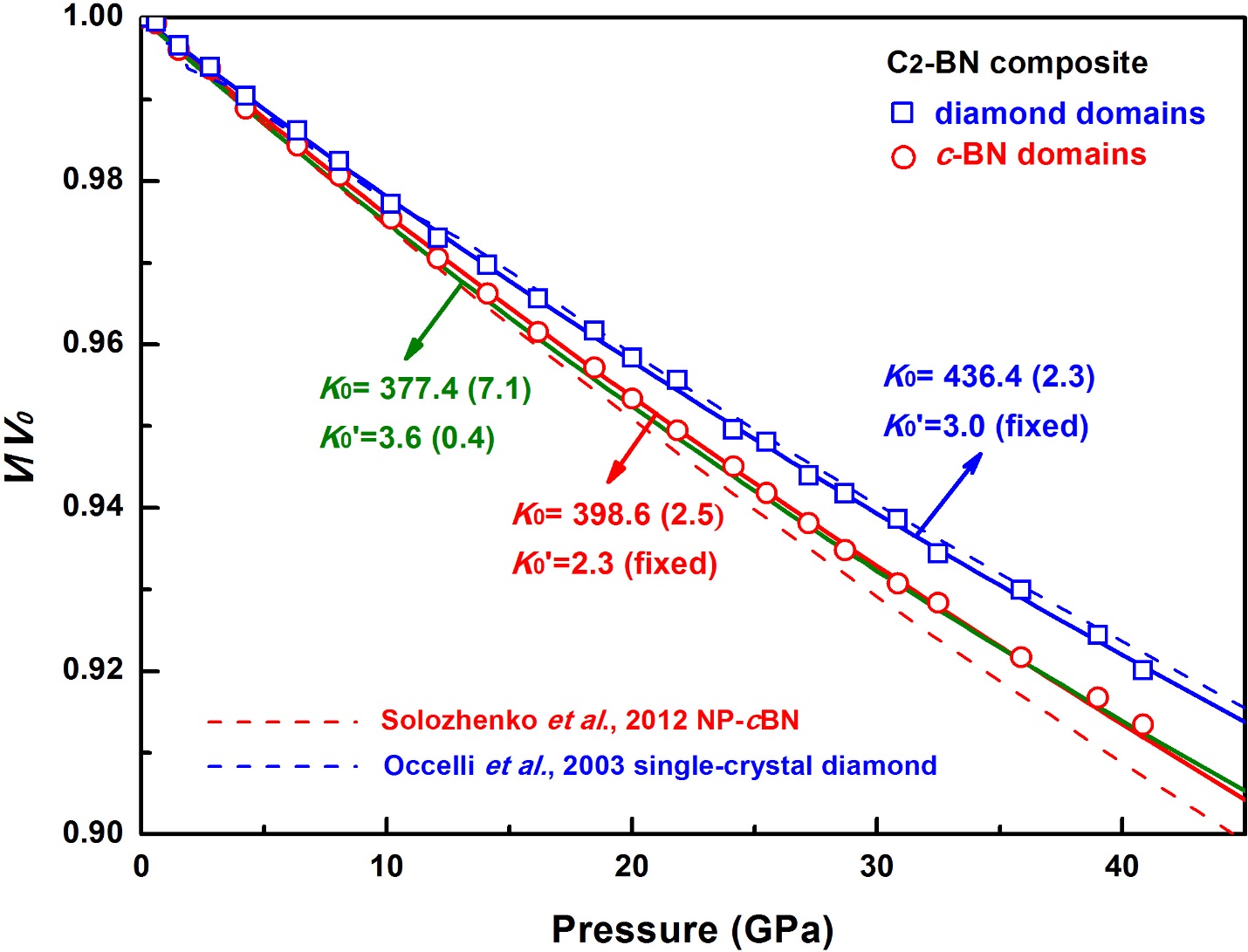


**Figure S10. Equation of state of C_2_-BN domains up to 40 GPa at 300 K.** Blue squares and red circles represent experimental points of diamond and *c*BN domains, while solid lines of the same color represent third-order Vinet equation-of-state (EOS) fits to the data where *K*_0_’ was not refined (fixed), see main text for details. A fit to the compression data of *c*BN domains where *K*_0_’ was allowed to refine is shown as the green solid line. For comparison, the equation of state for natural single-crystal diamond [Occelli *et al.* 2003] is shown by the blue dashed line and for nanopolycrystalline (NP) *c*BN [Solozhenko *et al.* 2012] by the red dashed line. Data from this study are given in Table S2.


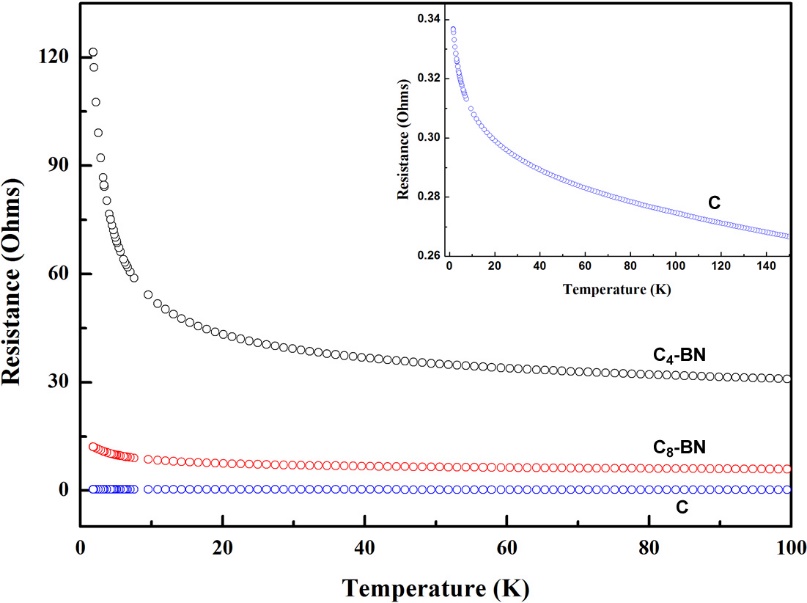


**Figure S11. A resistance-versus-temperature curve for C_4_-BN, C_8_-BN and C samples produced at 7.5 GPa and 2273 K.**

**Table S1. Comparison of the activation energy (*E_g_*) over different temperature ranges for boron-doped diamond, C_2_-BN, and BNC thin films.**

| Reference | composition | *E*_g_ (meV) | |
| --- | --- | --- | --- |
|  |  | 100-50 K | 10-1.8 K |
| **1**^[1]^ | B-diamond (0.1 at%) | 64.63 | 8.67 |
| **2**^[1]^ | B-diamond (1-1.5 at%) | 43.27 | 0.19 |
| **3**^[1]^ | C_2_-BN | 6.21 | 0.94 |
| **4**^[2]^ | BNC film | 9.09 | / |

*E*_g_ is the activation energy of dopant.

References: [1] This study; [2] Ci *et al*. (2010)

**Table S2. Pressure-Volume data* for *c*BN and diamond domains in C_2_-BN nanocomposite.**

| **Pressure** | ***cBN*** | **diamond** |
| --- | --- | --- |
| (GPa) | V (Å^3^) | V (Å^3^) |
| 0.6 | 47.27 | 45.30 |
| 1.5 | 47.12 | 45.17 |
| 2.8 | 47.01 | 45.05 |
| 4.3 | 46.77 | 44.89 |
| 6.34 | 46.56 | 44.70 |
| 8.1 | 46.39 | 44.53 |
| 10.2 | 46.14 | 44.29 |
| 12.1 | 45.91 | 44.10 |
| 14.1 | 45.70 | 43.95 |
| 16.2 | 45.48 | 43.76 |
| 18.5 | 45.27 | 43.58 |
| 20.1 | 45.10 | 43.43 |
| 21.9 | 44.91 | 43.31 |
| 24.1 | 44.70 | 43.04 |
| 25.5 | 44.55 | 42.97 |
| 27.2 | 44.37 | 42.78 |
| 28.7 | 44.22 | 42.68 |
| 30.9 | 44.02 | 42.54 |
| 32.5 | 43.91 | 42.35 |
| 35.9 | 43.60 | 42.15 |
| 39.0 | 43.36 | 41.90 |
| 40.8 | 43.21 | 41.70 |

*Standard deviation in pressure is nominally ±0.1 GPa, and ±0.01 Å^3^ in volume.

**Supplementary References**

Zhang, W. et al. Epitaxy on Diamond by Chemical Vapor Deposition: A Route to High-Quality Cubic Boron Nitride for Electronic Applications. *Adv. Mater.* 16, 1405-1408 (2004).

Zhang, X. et al. Epitaxy of cubic boron nitride on (001)-oriented diamond. *Nat. Mater*. 2, 312-315 (2003).

Liu, J., Huang, J., Zhou, H. & Antonietti, M. Uniform graphitic carbon nitride nanorod for efficient photocatalytic hydrogen evolution and sustained photoenzymatic catalysis. *ACS Appl. Mat. Interfaces* **6**, 8434-8440 (2014).

Ahmed, M. H., Byrne, J. A., McLaughlin, J., Elhissi, A. & Ahmed, W. Comparison between FTIR and XPS characterization of amino acid glycine adsorption onto diamond-like carbon (DLC) and silicon doped DLC. *Appl. Surf. Sci.* **273**, 507-514 (2013).

Ci, L. *et al.* Atomic layers of hybridized boron nitride and graphene domains. *Nat. Mater.* **9**, 430-435 (2010).

Ekimov, E.A. *et al*., Superconductivity in diamond. *Nature*, **428**, 542-545, (2004).

Occelli, F., Loubeyre, P. & LeToullec, R. Properties of diamond under hydrostatic pressures up to 140 GPa. *Nat. Mater.* **2**, 151-154 (2003).

Solozhenko, V. L., Kurakevych, O. O. & Le Godec, Y. Creation of Nanostuctures by Extreme Conditions: High-Pressure Synthesis of Ultrahard Nanocrystalline Cubic Boron Nitride. *Adv. Mater.* **24**, 1540-1544 (2012).

Takano Y., *et al.* Superconductivity in diamond thin film well above liquid helium temperature, *Appl*. *Phys. Lett.* **85**, 2851-2853, 2004.
